# Diniconazole

**DOI:** 10.1107/S160053681004804X

**Published:** 2010-11-24

**Authors:** Zhi-Qiang Xiong, Jin-Zhu Chen, Shi-He Wen, Xu-Liang Nie

**Affiliations:** a Department of Chemistry, Jiangxi Agricultural University, Nanchang 330045, People’s Republic of China; b Instrumental Analysis Center, Nanchang Hangkong University, Nanchang 330063, People’s Republic of China

## Abstract

The asymmetric unit of the title compound [systematic name: (*E*)-1-(2,4-dichloro­phen­yl)-4,4-dimethyl-2-(1*H*-1,2,4-triazol-1-yl)pent-1-en-3-ol], C_15_H_17_Cl_2_N_3_O, contains two mol­ecules in which the dihedral angles between the triazole and benzene rings are 9.4 (2) and 35.0 (2)°. In the crystal, the mol­ecules are linked by O—H⋯N hydrogen bonds, forming *C*(7) chains propagating in [010].

## Related literature

For background to the use of diniconazole as a fungicide, see: Sumitomo Chemical (1984 ▶). For further synthetic details, see: Xia *et al.* (2001 ▶).
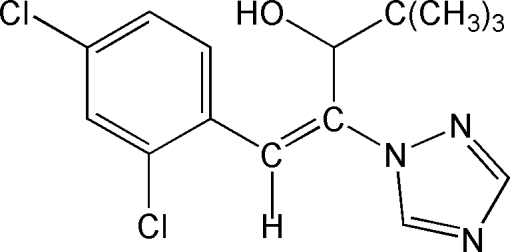


## Experimental

### 

#### Crystal data

C_15_H_17_Cl_2_N_3_O
*M* *_r_* = 326.22
Monoclinic, 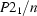

*a* = 7.2321 (15) Å
*b* = 20.248 (4) Å
*c* = 22.449 (5) Å
β = 96.072 (2)°
*V* = 3268.8 (12) Å^3^
*Z* = 8
Mo *K*α radiation
μ = 0.40 mm^−1^
*T* = 296 K
0.22 × 0.18 × 0.07 mm

#### Data collection

Bruker SMART APEX CCD diffractometer
Absorption correction: multi-scan (*SADABS*; Bruker, 1998 ▶) *T*_min_ = 0.917, *T*_max_ = 0.973
24930 measured reflections
6074 independent reflections
4232 reflections with *I* > 2σ(*I*)
*R*_int_ = 0.028

#### Refinement

*R*[*F* ^2^ > 2σ(*F* ^2^)] = 0.057
*wR*(*F* ^2^) = 0.179
*S* = 1.02
6074 reflections
391 parameters
1 restraint
H atoms treated by a mixture of independent and constrained refinement
Δρ_max_ = 1.06 e Å^−3^
Δρ_min_ = −0.53 e Å^−3^

### 

Data collection: *SMART* (Bruker, 1998 ▶); cell refinement: *SAINT* (Bruker, 1998 ▶); data reduction: *SAINT*; program(s) used to solve structure: *SHELXS97* (Sheldrick, 2008 ▶); program(s) used to refine structure: *SHELXL97* (Sheldrick, 2008 ▶); molecular graphics: *SHELXTL* (Sheldrick, 2008 ▶); software used to prepare material for publication: *SHELXTL*.

## Supplementary Material

Crystal structure: contains datablocks global, I. DOI: 10.1107/S160053681004804X/hb5745sup1.cif

Structure factors: contains datablocks I. DOI: 10.1107/S160053681004804X/hb5745Isup2.hkl

Additional supplementary materials:  crystallographic information; 3D view; checkCIF report

## Figures and Tables

**Table 1:** Hydrogen-bond geometry (Å, °)

*D*—H⋯*A*	*D*—H	H⋯*A*	*D*⋯*A*	*D*—H⋯*A*
O1—H1⋯N6^i^	0.82	2.04	2.844 (3)	165
O2—H2⋯N3^ii^	0.82	2.01	2.812 (4)	166

Symmetry codes: (i) 
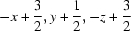
; (ii) 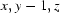.

**Fig. 1.**
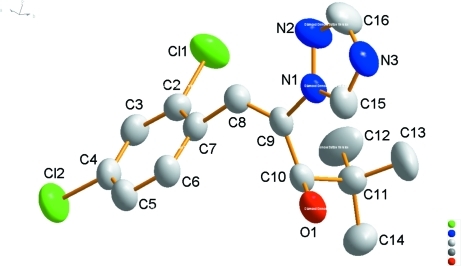
The molecular structure of (I), showing displacement ellipsoids at the 40% probability level.

**Fig. 2.**
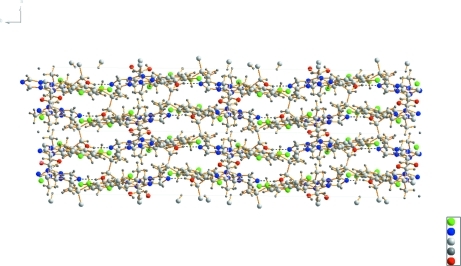
The crystal packing of (I), showing hydrogen-bonded chains; H-bonds are shown as dashed lines.

## supplementary crystallographic information

### Experimental

The synthesis process of diniconazole is carried out by the three steps of condensing, using a-triazolylpinacolone and 2,4-dichlorobenzaldehyde for material shifting and reduing, using a modified Xia reaction (Xia *et al.*, 2001). Diniconazole was recrystallized from ethanol, and colourless blocks were obtained by slow concentration of a water/ethanol solution. m.p. 134–138 °C (m.p.134–156°C). Anal. Calcd. For C15H17Cl2N3O (%) (Mr = 326.22): C, 55.23; H, 5.25; N, 12.88. Found (%): C, 55.21; H, 5.36; N, 12.90.

### Refinement

All H atoms were included in calculated positions and refined as riding atoms, with C—H = 0.93–0.96 Å, O—H = 0.82 Å and N—H = 0.86–0.89 Å, with *U*_iso_(H) = 1.5 *U*_eq_(C) for methyl H atoms and 1.2 *U*_eq_(C) for all other H atoms.

### Crystal data

**Table:**

C_15_H_17_Cl_2_N_3_O	*F*(000) = 1360
*M**_r_* = 326.22	*D*_x_ = 1.326 Mg m^−^^3^
Monoclinic, *P*2_1_/*n*	Mo *K*α radiation, λ = 0.71073 Å
Hall symbol: -P 2yn	Cell parameters from 6731 reflections
*a* = 7.2321 (15) Å	θ = 2.9–23.7°
*b* = 20.248 (4) Å	µ = 0.40 mm^−^^1^
*c* = 22.449 (5) Å	*T* = 296 K
β = 96.072 (2)°	Block, colourless
*V* = 3268.8 (12) Å^3^	0.22 × 0.18 × 0.07 mm
*Z* = 8	

### Data collection

**Table:**

Bruker SMART APEX CCD diffractometer	6074 independent reflections
Radiation source: fine-focus sealed tube	4232 reflections with *I* > 2σ(*I*)
graphite	*R*_int_ = 0.028
φ and ω scans	θ_max_ = 25.5°, θ_min_ = 2.2°
Absorption correction: multi-scan (*SADABS*; Bruker, 1998)	*h* = −8→8
*T*_min_ = 0.917, *T*_max_ = 0.973	*k* = −24→24
24930 measured reflections	*l* = −27→27

### Refinement

**Table:**

Refinement on *F*^2^	Primary atom site location: structure-invariant direct methods
Least-squares matrix: full	Secondary atom site location: difference Fourier map
*R*[*F*^2^ > 2σ(*F*^2^)] = 0.057	Hydrogen site location: inferred from neighbouring sites
*wR*(*F*^2^) = 0.179	H atoms treated by a mixture of independent and constrained refinement
*S* = 1.02	*w* = 1/[σ^2^(*F*_o_^2^) + (0.0857*P*)^2^ + 2.8116*P*] where *P* = (*F*_o_^2^ + 2*F*_c_^2^)/3
6074 reflections	(Δ/σ)_max_ < 0.001
391 parameters	Δρ_max_ = 1.06 e Å^−^^3^
1 restraint	Δρ_min_ = −0.53 e Å^−^^3^

### Special details

**Table:**

Geometry. All e.s.d.'s (except the e.s.d. in the dihedral angle between two l.s. planes) are estimated using the full covariance matrix. The cell e.s.d.'s are taken into account individually in the estimation of e.s.d.'s in distances, angles and torsion angles; correlations between e.s.d.'s in cell parameters are only used when they are defined by crystal symmetry. An approximate (isotropic) treatment of cell e.s.d.'s is used for estimating e.s.d.'s involving l.s. planes.
Refinement. Refinement of *F*^2^ against ALL reflections. The weighted *R*-factor *wR* and goodness of fit *S* are based on *F*^2^, conventional *R*-factors *R* are based on *F*, with *F* set to zero for negative *F*^2^. The threshold expression of *F*^2^ > σ(*F*^2^) is used only for calculating *R*-factors(gt) *etc*. and is not relevant to the choice of reflections for refinement. *R*-factors based on *F*^2^ are statistically about twice as large as those based on *F*, and *R*- factors based on ALL data will be even larger.

### Fractional atomic coordinates and isotropic or equivalent isotropic displacement parameters (Å^2^)

**Table:**

	*x*	*y*	*z*	*U*_iso_*/*U*_eq_	
Cl2	0.65600 (18)	0.67176 (5)	0.77688 (5)	0.0814 (4)	
Cl3	0.70579 (19)	0.44904 (6)	1.11219 (5)	0.0883 (4)	
Cl1	0.5999 (2)	0.83181 (5)	0.96433 (4)	0.0888 (4)	
Cl4	0.82764 (18)	0.21724 (9)	1.22070 (6)	0.1187 (6)	
N4	0.8131 (4)	0.43310 (12)	0.90776 (11)	0.0446 (6)	
C17	0.7857 (5)	0.37039 (19)	1.10971 (15)	0.0576 (9)	
C22	0.8479 (5)	0.34644 (17)	1.05758 (14)	0.0492 (8)	
C19	0.8393 (5)	0.2666 (2)	1.15758 (18)	0.0713 (12)	
C21	0.9102 (5)	0.28044 (19)	1.05634 (16)	0.0603 (9)	
C20	0.9004 (5)	0.2411 (2)	1.10768 (19)	0.0701 (11)	
H20	0.9365	0.1970	1.1072	0.084*	
C18	0.7834 (5)	0.3313 (2)	1.16074 (16)	0.0705 (11)	
H18	0.7452	0.3484	1.1959	0.085*	
O2	0.7417 (4)	0.29529 (11)	0.87882 (10)	0.0582 (6)	
H2	0.7670	0.2567	0.8872	0.087*	
C24	0.7835 (4)	0.38187 (14)	0.95063 (13)	0.0425 (7)	
C25	0.6605 (5)	0.32546 (15)	0.92710 (13)	0.0459 (7)	
H25	0.6629	0.2926	0.9592	0.055*	
N6	0.8689 (4)	0.48664 (14)	0.82749 (12)	0.0600 (8)	
N5	0.7781 (4)	0.49744 (13)	0.91967 (12)	0.0577 (8)	
C30	0.8680 (5)	0.42838 (17)	0.85327 (14)	0.0536 (8)	
H30	0.9012	0.3891	0.8357	0.064*	
C26	0.4538 (5)	0.34365 (17)	0.90934 (15)	0.0539 (8)	
C23	0.8597 (5)	0.39100 (16)	1.00591 (14)	0.0490 (8)	
H23	0.9283	0.4296	1.0132	0.059*	
C31	0.8128 (6)	0.52653 (17)	0.87023 (17)	0.0631 (10)	
H31	0.7997	0.5719	0.8648	0.076*	
C29	0.4280 (5)	0.38712 (18)	0.85297 (16)	0.0620 (9)	
H29A	0.4925	0.3677	0.8221	0.093*	
H29B	0.4774	0.4304	0.8623	0.093*	
H29C	0.2980	0.3905	0.8393	0.093*	
C28	0.3795 (6)	0.3783 (3)	0.96174 (19)	0.0833 (13)	
H28A	0.2479	0.3849	0.9532	0.125*	
H28B	0.4399	0.4203	0.9681	0.125*	
H28C	0.4037	0.3517	0.9971	0.125*	
C27	0.3483 (6)	0.2788 (2)	0.8956 (2)	0.0816 (13)	
H27A	0.2184	0.2880	0.8858	0.122*	
H27B	0.3644	0.2505	0.9300	0.122*	
H27C	0.3959	0.2573	0.8623	0.122*	
O1	0.4857 (4)	1.04993 (11)	0.77059 (10)	0.0589 (6)	
H1	0.5461	1.0329	0.7457	0.088*	
C10	0.4536 (5)	1.00280 (15)	0.81480 (14)	0.0478 (8)	
H10	0.4444	0.9597	0.7948	0.057*	
C9	0.6143 (4)	0.99840 (14)	0.86293 (14)	0.0440 (7)	
C7	0.6734 (4)	0.87704 (15)	0.85638 (14)	0.0466 (7)	
C2	0.6358 (5)	0.82160 (16)	0.88971 (14)	0.0500 (8)	
C8	0.6993 (5)	0.94335 (15)	0.88313 (15)	0.0484 (8)	
H8	0.7829	0.9470	0.9174	0.058*	
C5	0.6972 (5)	0.80365 (17)	0.77202 (16)	0.0564 (9)	
H5	0.7174	0.7976	0.7322	0.068*	
C4	0.6598 (5)	0.75077 (15)	0.80720 (16)	0.0533 (8)	
C3	0.6285 (5)	0.75888 (16)	0.86631 (16)	0.0549 (8)	
H3	0.6031	0.7228	0.8897	0.066*	
C6	0.7044 (5)	0.86592 (16)	0.79699 (15)	0.0532 (8)	
H6	0.7307	0.9017	0.7734	0.064*	
C11	0.2617 (5)	1.01600 (18)	0.83808 (16)	0.0569 (9)	
C14	0.1120 (6)	1.0049 (2)	0.7858 (2)	0.0780 (12)	
H14A	0.1323	1.0345	0.7537	0.117*	
H14B	−0.0082	1.0132	0.7987	0.117*	
H14C	0.1178	0.9601	0.7721	0.117*	
C12	0.2327 (6)	0.9669 (3)	0.8877 (2)	0.1003 (17)	
H12A	0.2481	0.9228	0.8735	0.150*	
H12B	0.1095	0.9720	0.8994	0.150*	
H12C	0.3222	0.9752	0.9216	0.150*	
C13	0.2417 (6)	1.0870 (2)	0.8601 (2)	0.0906 (15)	
H13A	0.1164	1.0941	0.8691	0.136*	
H13B	0.2702	1.1173	0.8295	0.136*	
H13C	0.3259	1.0940	0.8956	0.136*	
N1	0.6761 (4)	1.05896 (12)	0.89188 (12)	0.0494 (7)	
N3	0.7609 (5)	1.16084 (14)	0.91014 (16)	0.0682 (9)	
N2	0.7569 (6)	1.05941 (16)	0.94929 (15)	0.0791 (10)	
C15	0.6829 (5)	1.12015 (16)	0.86994 (17)	0.0593 (9)	
H15	0.6380	1.1322	0.8311	0.071*	
C16	0.8031 (7)	1.1211 (2)	0.9574 (2)	0.0840 (13)	
H16	0.8614	1.1367	0.9936	0.101*	
H21	1.0000 (13)	0.2641 (5)	1.0193 (4)	−0.050 (2)*	

### Atomic displacement parameters (Å^2^)

**Table:**

	*U*^11^	*U*^22^	*U*^33^	*U*^12^	*U*^13^	*U*^23^
Cl2	0.1121 (9)	0.0410 (5)	0.0934 (8)	0.0038 (5)	0.0210 (6)	−0.0089 (5)
Cl3	0.1113 (9)	0.0765 (7)	0.0782 (7)	0.0223 (6)	0.0151 (6)	−0.0110 (5)
Cl1	0.1515 (12)	0.0683 (6)	0.0495 (5)	−0.0040 (7)	0.0248 (6)	0.0095 (5)
Cl4	0.0886 (9)	0.1778 (15)	0.0906 (9)	0.0219 (9)	0.0131 (7)	0.0851 (10)
N4	0.0555 (16)	0.0405 (14)	0.0382 (13)	−0.0042 (12)	0.0067 (12)	0.0008 (11)
C17	0.056 (2)	0.069 (2)	0.0459 (18)	−0.0022 (17)	0.0006 (16)	−0.0012 (16)
C22	0.0500 (18)	0.059 (2)	0.0368 (16)	−0.0066 (15)	−0.0038 (14)	0.0024 (14)
C19	0.053 (2)	0.101 (3)	0.058 (2)	0.005 (2)	−0.0044 (18)	0.033 (2)
C21	0.057 (2)	0.062 (2)	0.059 (2)	0.0051 (17)	−0.0058 (17)	0.0132 (17)
C20	0.063 (2)	0.072 (3)	0.073 (3)	0.012 (2)	−0.002 (2)	0.026 (2)
C18	0.060 (2)	0.110 (4)	0.0413 (19)	−0.001 (2)	0.0067 (17)	0.006 (2)
O2	0.0883 (18)	0.0394 (12)	0.0486 (13)	0.0003 (12)	0.0155 (12)	−0.0052 (10)
C24	0.0518 (18)	0.0394 (16)	0.0367 (15)	0.0001 (13)	0.0060 (13)	0.0019 (12)
C25	0.064 (2)	0.0397 (16)	0.0340 (15)	−0.0045 (14)	0.0038 (14)	0.0038 (12)
N6	0.080 (2)	0.0554 (17)	0.0465 (16)	−0.0132 (15)	0.0149 (15)	0.0077 (13)
N5	0.084 (2)	0.0400 (15)	0.0513 (16)	−0.0028 (14)	0.0159 (15)	0.0001 (12)
C30	0.069 (2)	0.0512 (19)	0.0422 (17)	−0.0085 (16)	0.0126 (16)	−0.0022 (14)
C26	0.058 (2)	0.057 (2)	0.0450 (18)	−0.0062 (16)	−0.0011 (16)	0.0065 (15)
C23	0.058 (2)	0.0473 (18)	0.0416 (17)	−0.0051 (15)	0.0029 (15)	−0.0006 (14)
C31	0.088 (3)	0.0420 (18)	0.060 (2)	−0.0079 (18)	0.014 (2)	0.0083 (16)
C29	0.066 (2)	0.059 (2)	0.059 (2)	0.0001 (18)	−0.0065 (18)	0.0087 (17)
C28	0.062 (2)	0.124 (4)	0.066 (3)	0.011 (2)	0.014 (2)	−0.001 (3)
C27	0.081 (3)	0.083 (3)	0.075 (3)	−0.035 (2)	−0.015 (2)	0.022 (2)
O1	0.0888 (18)	0.0460 (13)	0.0459 (13)	0.0125 (12)	0.0254 (12)	0.0075 (10)
C10	0.065 (2)	0.0362 (16)	0.0441 (17)	0.0056 (14)	0.0131 (16)	0.0008 (13)
C9	0.0526 (18)	0.0358 (15)	0.0464 (17)	0.0004 (13)	0.0187 (15)	0.0000 (13)
C7	0.0503 (18)	0.0379 (16)	0.0523 (18)	0.0058 (14)	0.0088 (15)	0.0058 (13)
C2	0.057 (2)	0.0462 (18)	0.0470 (18)	0.0035 (15)	0.0052 (15)	0.0076 (14)
C8	0.0511 (19)	0.0426 (17)	0.0522 (18)	−0.0008 (14)	0.0092 (15)	0.0019 (14)
C5	0.070 (2)	0.0478 (18)	0.0539 (19)	0.0079 (16)	0.0181 (17)	0.0012 (15)
C4	0.058 (2)	0.0369 (17)	0.065 (2)	0.0070 (15)	0.0086 (17)	0.0001 (15)
C3	0.064 (2)	0.0380 (17)	0.063 (2)	0.0019 (15)	0.0069 (17)	0.0126 (15)
C6	0.066 (2)	0.0404 (17)	0.0561 (19)	0.0059 (15)	0.0193 (17)	0.0100 (15)
C11	0.056 (2)	0.064 (2)	0.0516 (19)	0.0035 (17)	0.0090 (16)	0.0021 (16)
C14	0.067 (3)	0.085 (3)	0.080 (3)	0.005 (2)	−0.003 (2)	0.001 (2)
C12	0.066 (3)	0.148 (5)	0.090 (3)	−0.004 (3)	0.025 (2)	0.046 (3)
C13	0.074 (3)	0.099 (3)	0.102 (3)	0.019 (3)	0.023 (3)	−0.038 (3)
N1	0.0616 (17)	0.0397 (14)	0.0490 (15)	−0.0004 (12)	0.0154 (13)	−0.0025 (11)
N3	0.089 (2)	0.0410 (16)	0.076 (2)	−0.0033 (15)	0.0162 (18)	−0.0121 (16)
N2	0.124 (3)	0.0556 (19)	0.0550 (19)	−0.0067 (19)	−0.0017 (19)	−0.0046 (15)
C15	0.076 (2)	0.0361 (17)	0.068 (2)	0.0006 (16)	0.0168 (19)	0.0019 (16)
C16	0.125 (4)	0.055 (2)	0.070 (3)	−0.005 (2)	−0.002 (3)	−0.017 (2)

### Geometric parameters (Å, °)

**Table:**

Cl2—C4	1.738 (3)	C27—H27B	0.9600
Cl3—C17	1.697 (4)	C27—H27C	0.9600
Cl1—C2	1.734 (3)	O1—C10	1.413 (4)
Cl4—C19	1.743 (4)	O1—H1	0.8200
N4—C30	1.329 (4)	C10—C9	1.504 (5)
N4—N5	1.359 (4)	C10—C11	1.557 (5)
N4—C24	1.446 (4)	C10—H10	0.9800
C17—C22	1.385 (5)	C9—C8	1.329 (4)
C17—C18	1.394 (5)	C9—N1	1.437 (4)
C22—C21	1.411 (5)	C7—C2	1.392 (4)
C22—C23	1.479 (4)	C7—C6	1.393 (5)
C19—C20	1.349 (6)	C7—C8	1.475 (4)
C19—C18	1.375 (6)	C2—C3	1.373 (5)
C21—C20	1.409 (5)	C8—H8	0.9300
C21—H21	1.157 (7)	C5—C4	1.374 (5)
C20—H20	0.9300	C5—C6	1.379 (5)
C18—H18	0.9300	C5—H5	0.9300
O2—C25	1.424 (4)	C4—C3	1.379 (5)
O2—H2	0.8200	C3—H3	0.9300
C24—C23	1.317 (4)	C6—H6	0.9300
C24—C25	1.508 (4)	C11—C12	1.524 (5)
C25—C26	1.550 (5)	C11—C14	1.527 (6)
C25—H25	0.9800	C11—C13	1.532 (6)
N6—C30	1.314 (4)	C14—H14A	0.9600
N6—C31	1.349 (5)	C14—H14B	0.9600
N5—C31	1.304 (4)	C14—H14C	0.9600
C30—H30	0.9300	C12—H12A	0.9600
C26—C28	1.516 (5)	C12—H12B	0.9600
C26—C27	1.534 (5)	C12—H12C	0.9600
C26—C29	1.536 (5)	C13—H13A	0.9600
C23—H23	0.9300	C13—H13B	0.9600
C31—H31	0.9300	C13—H13C	0.9600
C29—H29A	0.9600	N1—C15	1.336 (4)
C29—H29B	0.9600	N1—N2	1.358 (4)
C29—H29C	0.9600	N3—C15	1.305 (5)
C28—H28A	0.9600	N3—C16	1.341 (5)
C28—H28B	0.9600	N2—C16	1.302 (5)
C28—H28C	0.9600	C15—H15	0.9300
C27—H27A	0.9600	C16—H16	0.9300
			
C30—N4—N5	109.3 (3)	C10—O1—H1	109.5
C30—N4—C24	129.9 (3)	O1—C10—C9	111.9 (3)
N5—N4—C24	120.8 (2)	O1—C10—C11	109.8 (3)
C22—C17—C18	121.9 (4)	C9—C10—C11	114.7 (3)
C22—C17—Cl3	119.9 (3)	O1—C10—H10	106.7
C18—C17—Cl3	118.2 (3)	C9—C10—H10	106.7
C17—C22—C21	118.8 (3)	C11—C10—H10	106.7
C17—C22—C23	120.0 (3)	C8—C9—N1	116.8 (3)
C21—C22—C23	121.1 (3)	C8—C9—C10	126.1 (3)
C20—C19—C18	122.1 (4)	N1—C9—C10	117.0 (3)
C20—C19—Cl4	120.2 (4)	C2—C7—C6	116.4 (3)
C18—C19—Cl4	117.7 (3)	C2—C7—C8	122.6 (3)
C20—C21—C22	118.4 (4)	C6—C7—C8	120.7 (3)
C20—C21—H21	120.4 (6)	C3—C2—C7	122.8 (3)
C22—C21—H21	119.3 (6)	C3—C2—Cl1	118.4 (2)
C19—C20—C21	120.8 (4)	C7—C2—Cl1	118.7 (3)
C19—C20—H20	119.6	C9—C8—C7	126.2 (3)
C21—C20—H20	119.6	C9—C8—H8	116.9
C19—C18—C17	118.0 (4)	C7—C8—H8	116.9
C19—C18—H18	121.0	C4—C5—C6	118.7 (3)
C17—C18—H18	121.0	C4—C5—H5	120.7
C25—O2—H2	109.5	C6—C5—H5	120.7
C23—C24—N4	116.7 (3)	C5—C4—C3	121.5 (3)
C23—C24—C25	127.5 (3)	C5—C4—Cl2	119.2 (3)
N4—C24—C25	115.7 (2)	C3—C4—Cl2	119.3 (3)
O2—C25—C24	108.4 (3)	C2—C3—C4	118.3 (3)
O2—C25—C26	111.7 (3)	C2—C3—H3	120.8
C24—C25—C26	115.3 (3)	C4—C3—H3	120.8
O2—C25—H25	107.0	C5—C6—C7	122.3 (3)
C24—C25—H25	107.0	C5—C6—H6	118.9
C26—C25—H25	107.0	C7—C6—H6	118.9
C30—N6—C31	102.0 (3)	C12—C11—C14	109.0 (4)
C31—N5—N4	102.1 (3)	C12—C11—C13	110.5 (4)
N6—C30—N4	110.9 (3)	C14—C11—C13	107.6 (3)
N6—C30—H30	124.5	C12—C11—C10	109.3 (3)
N4—C30—H30	124.5	C14—C11—C10	107.5 (3)
C28—C26—C27	110.1 (3)	C13—C11—C10	112.8 (3)
C28—C26—C29	110.6 (3)	C11—C14—H14A	109.5
C27—C26—C29	108.1 (3)	C11—C14—H14B	109.5
C28—C26—C25	108.7 (3)	H14A—C14—H14B	109.5
C27—C26—C25	107.1 (3)	C11—C14—H14C	109.5
C29—C26—C25	112.2 (3)	H14A—C14—H14C	109.5
C24—C23—C22	126.9 (3)	H14B—C14—H14C	109.5
C24—C23—H23	116.6	C11—C12—H12A	109.5
C22—C23—H23	116.6	C11—C12—H12B	109.5
N5—C31—N6	115.7 (3)	H12A—C12—H12B	109.5
N5—C31—H31	122.1	C11—C12—H12C	109.5
N6—C31—H31	122.1	H12A—C12—H12C	109.5
C26—C29—H29A	109.5	H12B—C12—H12C	109.5
C26—C29—H29B	109.5	C11—C13—H13A	109.5
H29A—C29—H29B	109.5	C11—C13—H13B	109.5
C26—C29—H29C	109.5	H13A—C13—H13B	109.5
H29A—C29—H29C	109.5	C11—C13—H13C	109.5
H29B—C29—H29C	109.5	H13A—C13—H13C	109.5
C26—C28—H28A	109.5	H13B—C13—H13C	109.5
C26—C28—H28B	109.5	C15—N1—N2	108.3 (3)
H28A—C28—H28B	109.5	C15—N1—C9	130.3 (3)
C26—C28—H28C	109.5	N2—N1—C9	121.1 (3)
H28A—C28—H28C	109.5	C15—N3—C16	102.4 (3)
H28B—C28—H28C	109.5	C16—N2—N1	102.6 (3)
C26—C27—H27A	109.5	N3—C15—N1	111.1 (3)
C26—C27—H27B	109.5	N3—C15—H15	124.4
H27A—C27—H27B	109.5	N1—C15—H15	124.4
C26—C27—H27C	109.5	N2—C16—N3	115.5 (4)
H27A—C27—H27C	109.5	N2—C16—H16	122.2
H27B—C27—H27C	109.5	N3—C16—H16	122.2
			
C18—C17—C22—C21	0.2 (5)	O1—C10—C9—C8	−130.0 (3)
Cl3—C17—C22—C21	−178.8 (3)	C11—C10—C9—C8	104.2 (4)
C18—C17—C22—C23	−175.9 (3)	O1—C10—C9—N1	53.3 (3)
Cl3—C17—C22—C23	5.2 (5)	C11—C10—C9—N1	−72.6 (3)
C17—C22—C21—C20	2.1 (5)	C6—C7—C2—C3	−0.1 (5)
C23—C22—C21—C20	178.1 (3)	C8—C7—C2—C3	−173.9 (3)
C18—C19—C20—C21	0.3 (6)	C6—C7—C2—Cl1	178.9 (3)
Cl4—C19—C20—C21	179.9 (3)	C8—C7—C2—Cl1	5.2 (5)
C22—C21—C20—C19	−2.4 (6)	N1—C9—C8—C7	−173.0 (3)
C20—C19—C18—C17	2.0 (6)	C10—C9—C8—C7	10.3 (5)
Cl4—C19—C18—C17	−177.6 (3)	C2—C7—C8—C9	−129.4 (4)
C22—C17—C18—C19	−2.2 (6)	C6—C7—C8—C9	57.1 (5)
Cl3—C17—C18—C19	176.7 (3)	C6—C5—C4—C3	0.4 (5)
C30—N4—C24—C23	131.4 (4)	C6—C5—C4—Cl2	−178.4 (3)
N5—N4—C24—C23	−51.0 (4)	C7—C2—C3—C4	−0.1 (5)
C30—N4—C24—C25	−51.3 (5)	Cl1—C2—C3—C4	−179.1 (3)
N5—N4—C24—C25	126.2 (3)	C5—C4—C3—C2	−0.1 (5)
C23—C24—C25—O2	−123.9 (3)	Cl2—C4—C3—C2	178.7 (3)
N4—C24—C25—O2	59.2 (3)	C4—C5—C6—C7	−0.5 (5)
C23—C24—C25—C26	110.0 (4)	C2—C7—C6—C5	0.4 (5)
N4—C24—C25—C26	−66.9 (3)	C8—C7—C6—C5	174.3 (3)
C30—N4—N5—C31	0.8 (4)	O1—C10—C11—C12	−177.4 (3)
C24—N4—N5—C31	−177.1 (3)	C9—C10—C11—C12	−50.4 (4)
C31—N6—C30—N4	0.5 (4)	O1—C10—C11—C14	64.5 (4)
N5—N4—C30—N6	−0.9 (4)	C9—C10—C11—C14	−168.6 (3)
C24—N4—C30—N6	176.8 (3)	O1—C10—C11—C13	−54.0 (4)
O2—C25—C26—C28	−177.3 (3)	C9—C10—C11—C13	72.9 (4)
C24—C25—C26—C28	−53.0 (4)	C8—C9—N1—C15	148.1 (4)
O2—C25—C26—C27	63.7 (3)	C10—C9—N1—C15	−34.9 (5)
C24—C25—C26—C27	−171.9 (3)	C8—C9—N1—N2	−25.6 (4)
O2—C25—C26—C29	−54.7 (4)	C10—C9—N1—N2	151.4 (3)
C24—C25—C26—C29	69.7 (4)	C15—N1—N2—C16	1.0 (5)
N4—C24—C23—C22	179.3 (3)	C9—N1—N2—C16	176.0 (3)
C25—C24—C23—C22	2.5 (6)	C16—N3—C15—N1	0.7 (4)
C17—C22—C23—C24	−125.7 (4)	N2—N1—C15—N3	−1.1 (4)
C21—C22—C23—C24	58.3 (5)	C9—N1—C15—N3	−175.5 (3)
N4—N5—C31—N6	−0.5 (5)	N1—N2—C16—N3	−0.6 (6)
C30—N6—C31—N5	0.0 (5)	C15—N3—C16—N2	0.0 (6)

### Hydrogen-bond geometry (Å, °)

**Table:**

*D*—H···*A*	*D*—H	H···*A*	*D*···*A*	*D*—H···*A*
O1—H1···N6^i^	0.82	2.04	2.844 (3)	165
O2—H2···N3^ii^	0.82	2.01	2.812 (4)	166

Symmetry codes: (i) −*x*+3/2, *y*+1/2, −*z*+3/2; (ii) *x*, *y*−1, *z*.
